# Estimating minimal important change of the National Institutes of health research task force impact score using computer adaptive measures: a secondary analysis of two randomized clinical trials in a military population with chronic pain

**DOI:** 10.1186/s12891-025-08378-5

**Published:** 2025-02-11

**Authors:** Diane M. Flynn, Larisa A. Burke, Alana D. Steffen, Jeffrey C. Ransom, Kira P. Orr, Honor M. McQuinn, Tyler J. Snow, Ardith Z. Doorenbos

**Affiliations:** 1 Interdisciplinary Pain Management Center, Madigan Army Medical Center, 9040 Jackson Avenue, Tacoma, WA 98431 USA; 2 College of Nursing, University of Illinois Chicago, 845 S. Damen Avenue, Chicago, IL 60612 USA; 3 The Geneva Foundation, 950 Broadway, Suite 307, Tacoma, WA 98402 USA; 4 Department of Anesthesiology and Pain Medicine, University of Washington School of Medicine, 1959 NE Pacific St., Campus Box 356540, Seattle, WA 98195 USA

**Keywords:** Chronic pain impact, PROMIS, Minimal important change

## Abstract

**Background:**

The National Institutes of Health (NIH) Research Task Force (RTF) on Research Standards for Chronic Low Back Pain impact score is a composite measure of Patient Reported Outcomes Measurement Information System (PROMIS) pain intensity, pain interference and physical function. PROMIS surveys are available in short-form and computer adaptive testing (CAT) formats. Minimal important change (MIC) can be estimated to determine if between-group differences are large enough to be important. To date, three anchor-based estimates of impact score MIC ranging from 3 to 7.5 have been published, and all were based on data collected using PROMIS short-form surveys. None used CAT versions of PROMIS surveys.

**Methods:**

Secondary analysis of data collected during the conduct of two randomized clinical trials of 6-week courses of nonpharmacological pain therapies. Research subjects were US active-duty service members referred to an interdisciplinary pain management center. Impact score was assessed at the beginning and end of treatment. The Patient Global Impression of Change (PGIC) questionnaire was administered at the end of treatment and asked respondents to report their status compared to the start of treatment using a 7-item categorical scale ranging from very much improved to very much worse. A PGIC response of “much” or “very much” improved defined important improvement. Receiver operating characteristic (ROC) curve analysis and predictive logistic regression models were used to estimate MIC for the full combined sample and stratified by study sample and baseline impact score. Measures of individual statistical change were also computed.

**Results:**

Overall, a decrease of 3 points in impact score was the estimated MIC (2.5 for ROC analysis and 3.4 for predictive modeling approach). Larger decreases in impact score were needed for participants with moderate and severe baseline pain impact to report important improvement. Thresholds for individual statistically significant change ranged from 6 to 14.

**Conclusions:**

Using data collected with CAT surveys, we calculated an MIC of 3 points for the NIH RTF impact score, and estimates ranged from 1.3 to 7.2 depending on the baseline impact score and statistical approach used. These findings are consistent with previous MIC estimates that were based on non-adaptive short form surveys and have implications for improving the accuracy of pain treatment response assessment.

**Registry information:**

Trial registration. ClinicalTrials.gov. Registry numbers: NCT03297905 (registered 9/29/17) and NCT04656340 (registered 11/30/20). Link to full applications: https://classic.clinicaltrials.gov/ct2/show/NCT03297905?titles=Determinants+of+Optimal+Dosage%26cntry=US%26draw=2%26rank=1; https://classic.clinicaltrials.gov/ct2/show/results/NCT04656340?titles=Complementary+and+Integrative+pain+therapies+and+functional+restoration+%28IMPPPORT%29%26draw=2%26rank=1. Patient enrollment dates: SMART: 17 March 2021, prospectively registered; IMPPPORT: 9 December 2015, retrospectively registered.

**Supplementary Information:**

The online version contains supplementary material available at 10.1186/s12891-025-08378-5.

## Background

The pain impact score (PIS)—variably referred to as the Impact Stratification Score [1–3], RTF impact score [4], Pain Impact Stratification Score [5], and Pain Impact Score [6]—is a composite measure of Patient-Reported Outcomes Measurement Information System (PROMIS) measures of pain intensity, pain interference, and physical function. The National Institutes of Health (NIH) Task Force on Research Standards for Chronic Low Back Pain (RTF) has endorsed the PIS as a tool to stratify the impact of musculoskeletal pain on the lives of those who experience it [1].

The Initiative on Methods, Measurement, and Pain Assessment in Clinical Trials (IMMPACT) consensus statement of 2020 states that “statistically significant evidence of a treatment’s efficacy in a clinical trial is insufficient to indicate that the magnitude of the treatment effect is clinically important” and that “evaluations of clinical importance must distinguish between determining whether the mean improvements are important to patients or whether the group differences between treatments in an RCT are clinically important” [7]. The statements distinguish between within-patient minimal clinically meaningful change and between group minimal clinically meaningful differences. Group level minimal clinically important change (MIC) estimates include anchor-based approaches that relate change scores on an instrument to an external criterion of important change. Distribution based approaches can be used to determine thresholds for individual statistically significant change [8, 9].

It has been argued that anchor-based MIC thresholds are not appropriate to identify responders to treatment because MIC estimates are averages derived from distributions of individual MICs and therefore may not reflect the perceived change by a given individual. For that reason, it is suggested that use of statistical measures of individual change also be conducted when attempting to identify responders to treatment [9].

To date, three anchor-based estimates of the PIS MIC have been published [2–4, 10]. The first published estimate was based on data collected from a rural population of adults with musculoskeletal pain, age 55 years or older, who completed the paper version of the PROMIS-29 questionnaire [4]. The authors recommended further studies with data collected from other populations, and advocated for use of computer-adaptive testing (CAT) versions of the PROMIS measures. CAT is a process by which sequential items in a questionnaire are based on previous responses in order to assess the outcome variable using the fewest possible questions. Compared with the PROMIS 4-item short-form questionnaires, CAT yields improved accuracy with a slightly higher survey burden averaging 4.7 items [11]. Yet all three previously published studies of the PIS MIC collected PIS data using paper or digital versions of the PROMIS short-form pain interference and physical function questionnaires; none collected data using CAT versions of these assessments.

## Methods

The primary aim of the study was to address this gap by estimating the PIS MIC from data collected using CAT versions of PROMIS measures of pain interference and physical function. The secondary aim was to explore the psychometric properties of the PIS. The data used for this secondary analysis were collected during two unrelated randomized clinical trials (RCTs) of nonpharmacological pain therapies provided to US active-duty Army, Navy, and Air Force service members. Both clinical trials were approved by the Madigan Army Medical Center institutional review board. The Integrative Modalities Plus Psychological, Physical, and Occupational Restorative Therapies (IMPPPORT) study enrolled 210 participants and was designed to determine if adding complementary and integrative health therapies enhanced the outcomes of an intensive functional restoration program (protocol #215050). The Complementary, Integrative, and Standard Rehabilitative Pain Therapies Pragmatic Trial with SMART Design (SMART study) enrolled 280 participants and was designed to determine the optimal sequence, duration, and combination of physical, occupational, and complementary and integrative health therapies for treatment of chronic, predominantly musculoskeletal pain (protocol #221011). The research protocols for both RCTs have been described in detail [12, 13].

### Study participants

Research participants for both RCTs were recruited from the population of US active-duty service members referred to the Madigan Army Medical Center Interdisciplinary Pain Management Center. Enrollment occurred between 9 December 2015 and 16 May 2018 for the IMPPPORT study and between 17 March 2021 and 9 September 2022 for the SMART study. Both studies’ inclusion criteria required active-duty military status and functional impairment due to pain. In addition, inclusion criteria for the IMPPPORT study required the ability to meet modest functional thresholds (stand up from and sit down on floor independently, walk or jog on a treadmill for at least 6 min, lift and/or carry at least 20 lbs.) to ensure participants’ capacity to engage in an intensive functional restoration program. Exclusion criteria for both studies were inability to commit the time required for treatment, surgery within the previous or following 6 months, unstable psychological condition(s) or being in the process of medical disability determination at the time of study enrollment. The combined enrolled population of both RCTs was 365; no participants were enrolled in both studies.

### Study procedures

For each of the original RCTs, potential participants were recruited through in-person discussion with a member of the research team immediately following a routine interdisciplinary pain management center visit. No compensation was offered for participation. Following informed consent, participants in each study were randomized to the first of two 3-week treatment stages. In both studies, participants were asked to provide measures at baseline, at the end of stage 1 and stage 2, and at 3- and 6-month post-treatment follow-ups. In addition, participants were asked at the end of stage 2 to report their perceived level of improvement relative to baseline.

### Study measures

#### Pain impact score (PIS) components

The PIS was originally described as the sum of the PROMIS 7-day average pain intensity (from 0 = “no pain” through 10 = “worst imaginable pain”), PROMIS pain interference short-form 4a v1.1 score (range: 4–20), and the reverse of the PROMIS physical function short-form 4a v.1.0 score (range: 4–20). This computation resulted in a PIS that ranged from 8 (least impact) to 50 (most impact) [4]. Our current analysis instead used the Defense and Veterans Pain Rating Scale (DVPRS) to determine 7-day average pain intensity and CAT versions of the PROMIS pain interference [14, 15] and physical function measures [16]. The DVPRS also has a range of 0 (“no pain”) to 10 (“as bad as it could be, nothing else matters”); uses a combination of numeric, color, facial expression, and word descriptors; and has been validated in military and veteran populations [17, 18]. The CAT versions of PROMIS report T-scores; thus, to compute the PIS, we took the additional step of converting the PROMIS T-scores into scores equivalent to the PROMIS short-form scores. T-score equivalents of each short-form score are published in the user manuals for PROMIS pain interference and physical function measures [19]. Steps for calculating pain impact score using PROMIS computer adaptive tests are described in Table 1.

**Table 1:** Calculation of pain impact score from PROMIS computer adaptive instruments

Physical function 4a - Adult v1.0			Pain interference 4a - Adult v1.1		
Raw score	T-score		Raw score	T-score	
	Low cut-off	High cut-off		Low cut-off	High cut-off
4	≥ 0	< 22.9	4	≥ 0	< 41.6
4	≥ 22.9	< 26.9	4	≥ 41.6	< 49.6
5	≥ 26.9	< 29.1	5	≥ 49.6	< 52.0
6	≥ 29.1	< 30.7	6	≥ 52.0	< 53.9
7	≥ 30.7	< 32.1	7	≥ 53.9	< 55.6
8	≥ 32.1	< 33.3	8	≥ 55.6	< 57.1
9	≥ 33.3	< 34.4	9	≥ 57.1	< 58.5
10	≥ 34.4	< 35.6	10	≥ 58.5	< 59.9
11	≥ 35.6	< 36.7	11	≥ 59.9	< 61.2
12	≥ 36.7	< 37.9	12	≥ 61.2	< 62.5
13	≥ 37.9	< 39.1	13	≥ 62.5	< 63.8
14	≥ 39.1	< 40.4	14	≥ 63.8	< 65.2
15	≥ 40.4	< 41.8	15	≥ 65.2	< 66.6
16	≥ 41.8	< 43.4	16	≥ 66.6	< 68.0
17	≥ 43.4	< 45.3	17	≥ 68.0	< 69.7
18	≥ 45.3	< 48.0	18	≥ 69.7	< 71.6
19	≥ 48.0	< 56.9	19	≥ 71.6	< 75.6
20	≥ 56.9	<=100	20	≥ 75.6	<= 100

Step 1. Convert pain interference and physical function CAT T-Scores to short-form raw scores using conversion tables above

Step 2: Reverse physical function raw score by subtracting it from 24

Step 3: Add raw score for pain interference and reversed raw score for physical function to the 7-day average pain intensity score (patient rating from 0 to 10 of average pain intensity in the past 7 days)

Example: **Average pain intensity** is 7. **Pain interference** T-score is 60.10, raw score equivalent is 11. **Physical function** T-score is 35.05, raw score equivalent is 10, reverse raw score is 24 − 10 = 14. **PIS** = 7 + 11 + 14 = 32

#### Additional PROMIS measures

In addition to the PIS components described above, this study’s outcome measures included the CAT versions of PROMIS sleep-related impairment, fatigue, depression, anxiety, and anger questionnaires [20–22].

#### Patient global impression of change

The Patient Global Impression of Change (PGIC) questionnaire [23] asks respondents to report their overall status compared to their status at the start of the research study, using the following 7-item categorical scale: “very much improved,” “much improved,” “minimally improved,” “no change,” “minimally worse,” “much worse,” “very much worse.” All participants in the SMART study were asked to complete the PGIC at the end of stage 2 of their study treatment and, in addition, to use the same 7-item scale to report perceived change in each of the 3 components of the PIS: (a) 7-day average pain intensity, (b) physical function (phrased as “ability to engage in physical activities”), and (c) pain interference (phrased as “ability to do day-to-day tasks and enjoy recreational and social activities”). The IMPPPORT study added collection of posttreatment PGIC questionnaires in June 2017 through an IRB-approved protocol modification, then collected PGIC data from the final 85 of 210 participants to enroll.

### Statistical methods

One recommended approach to anchor-based MIC estimation is using receiver operating characteristic (ROC) curves to determine the optimal cutpoint between change in an outcome measure and the corresponding self-report of perceived improvement, such as “much improved.” [8] There is no consensus on which PGIC response corresponds with both “minimal” and “important” change, but previous PIS MIC analyses judged responses of “little” improvement to be insufficient, and used cutpoints of at least “moderately” or “much” improved to indicate a MIC [2, 4, 10]. Unlike previous studies of PIS MIC estimates, the version of PGIC used in the current analysis did not include the category of a “little” improvement; the closest match is “minimally improved”. In an effort to be as consistent as possible with previous research, we considered “minimally improved” to be insufficient to be important to most respondents and used “much improved” as the cutpoint for MIC.

A disadvantage of the ROC approach is that the MIC estimate it yields will be an under- or overestimate if the percentage who report important improvement is not equal to 50%.^8^ When this percentage is considerably larger or smaller than 50%, a preferred anchor-based method of estimating MIC is predictive modeling using logistic regression. With this method, the dichotomous variable indicating improvement is the outcome, the change score is the predictor, and the MIC value is the change score associated with a likelihood ratio of 1 [24].

Baseline characteristics were assessed separately for each study population (IMPPPORT and SMART) and compared statistically with t-test, chi-square, and Fisher’s exact tests where applicable. Because the PGIC items were used as anchors for MIC, we compared the relationship between the mean change in PIS (pre to post) and the PGIC items for each study population by calculating bivariate correlations stratified by study and graphing the relationships with box plots. PGIC response categories were collapsed into bivariate categories for the MIC analysis, which compared either “much improved” or “very much improved” to other categories representing less or no improvement or worsening status. The primary anchor for the MIC analysis was change in overall status. We also conducted MIC analysis for the SMART study using the PGIC items on pain intensity, physical function, and pain interference as anchors. For the ROC approach, the CUTPT module in Stata (StataCorp LLC, College Station, Texas, USA) [25] was used to estimate anchor-based MIC, and the Euclidean distance method (point nearest to 0,1) was used to estimate the cutpoint that maximized both sensitivity and specificity [26]. For the predictive modeling approach, the MIC values were calculated using a spreadsheet included as a supplement to Terluin et al. (2015) [24]. Values were adjusted for the proportion of patients reporting important improvement. Statistical measures used to assess significance of individual change included Standard Deviation Index, Standard Error of Measurement, Standard Error of Estimate, Standard Error of Prediction, Reliable Change Index and the Coefficient of Repeatability [9].

Responsiveness is the ability of a measure to detect clinical changes [2]. The responsiveness of the PIS was assessed by the presence of significant (at *P* <.05) mean change pre to post intervention as determined by paired t-tests and by the area under the curve (AUC) values from the ROC curve analysis. An AUC higher than 0.70 was considered responsive as it indicates a high correlation between change in PIS and patient reported change. We assessed MICs and responsiveness of the PIS for the combined sample, for each study separately, and stratified by baseline impact score.

## Results

The sample for this secondary analysis included 61 participants from the IMPPPORT study and 192 participants from the SMART study who had completed the posttreatment PGIC and had both pre- and posttreatment PIS scores. The demographic characteristics, clinical characteristics, and PGIC responses of both study populations are shown in Table 2. The combined population (*n* = 253) was predominantly married male Army service members over 25 years of age with at least some college education. Compared to the IMPPPORT study population, the SMART study population was older, of higher military rank, were less likely to have their principal pain condition coded as musculoskeletal type and had a higher (worse) baseline PIS; higher (worse) PROMIS scores for baseline pain interference, depression, anger, and sleep impairment; and a lower (worse) PROMIS score for physical function. For both studies, the most common responses for improvement in overall status were “minimally improved” (36%) and “much improved” (32%); while 6% reported “very much improved” and 24% reported either no change or a worsening from baseline.

**Table 2:** Participant demographic and clinical characteristics at Baseline and impressions of Change following treatment

	IMPPPORT MIC population (*n* = 61)		SMART MIC population (*n* = 192)		Sig diff
Age (years)	*n*	%	*n*	%	*
<25	17	27.9%	13	6.8%	
25–34	21	34.4%	88	45.8%	
≥35	23	37.7%	91	47.4%	
Male^a^	52	85.2%	150	78.1%	
Married/partnered	44	72.1%	136	70.8%	
High school/GED only^b^	19	31.1%	45	23.4%	
Active-duty Army	52	85.2%	158	82.3%	
Military rank: junior enlisted	25	41.0%	50	26.0%	*
Pain type					*
Musculoskeletal (ICD-10 M00-M99)	45	86.3%	122	64.5%	
Nerves and senses (ICD-10 G00-H95, includes G89.xx chronic pain codes)	6	9.8%	60	31.3%	
Other	2	3.9%	10	5.2%	
PROMIS mean T-scores ^d^	Mean	SD	Mean	SD	
Pain interference	61.3	4.4	64.5	5.3	*
Physical function	41.6	5.1	39.7	5.0	*
Depression	50.0	9.2	54.7	10.4	*
Anxiety	53.9	10.4	56.7	9.9	
Anger	50.4	10.8	56.6	11.8	*
Fatigue	57.4	10.1	59.9	9.3	
Sleep-related impairment	58.7	9.6	61.9	9.2	*
Pain intensity	5.5	1.2	5.6	1.5	
Pain impact score	26.4	6.0	29.9	7.0	*
Overall status					
Very much improved	7	11%	8	4%	
Much improved	21	34%	60	32%	
Minimally improved	21	34%	69	36%	
No change	7	11%	33	17%	
Minimally worse	5	8%	12	6%	
Much worse	0	0%	7	4%	

^a^ The demographic questionnaires offered only two choices for sex: male or female

^b^ One participant was missing data on education in IMPPPORT study

^c^ Pain type was missing for 10 IMPPPORT subjects

^d^ PROMIS measures are reported by T-scores, with a range of 0-100, US reference population mean of 50.0 and SD equal to 10. For physical function, lower scores indicate worse functioning relative to higher scores. For all other PROMIS measures listed, higher scores indicate worse scores relative to lower scores

* Significance was set at *P* <.05

**Fig. 1:**
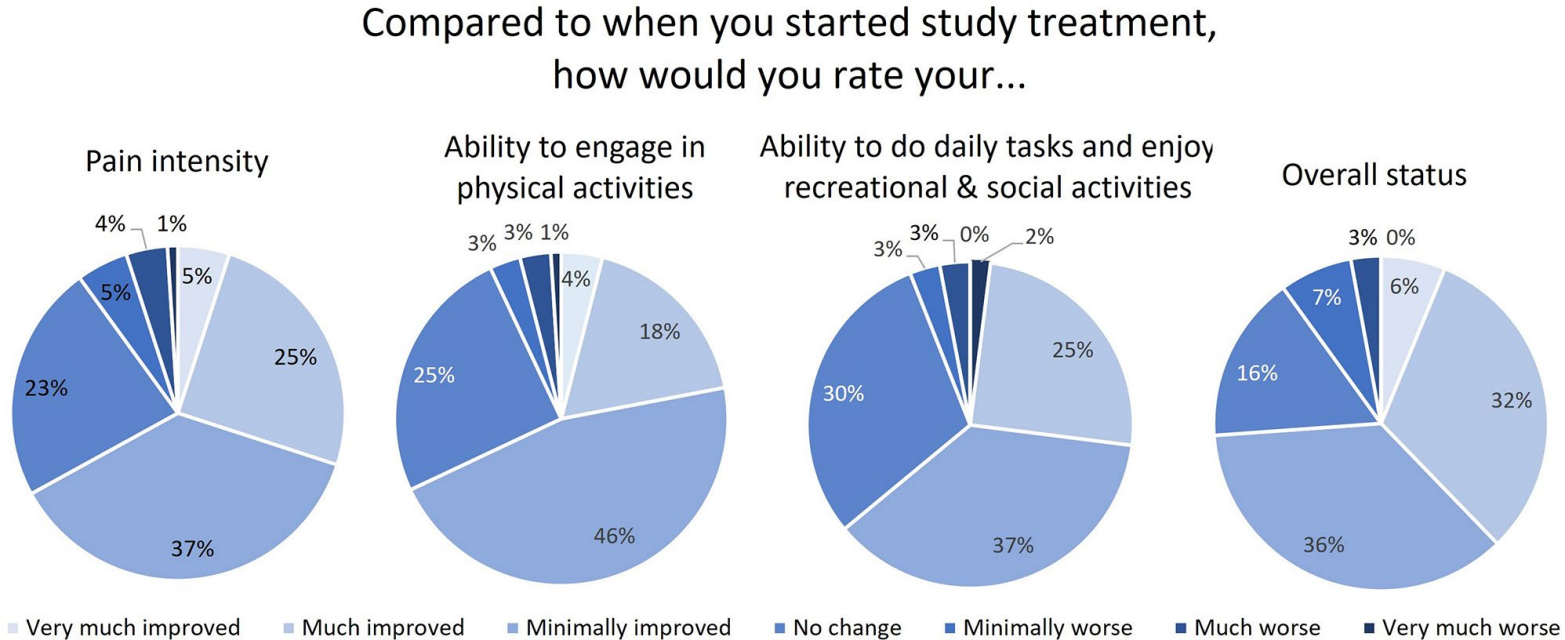
Patient impression of change following treatment. Distribution of 7 offered responses ranging from “very much improved” to “very much worse” for each anchor: pain intensity, physical function, pain interference and overall status

Figure 1 displays the SMART study participants’ responses to all four PGIC items. Reported impression of change varied across items. Results for Fisher’s exact tests found statistical differences between reported overall change compared to physical activity (*P* =.01) and ability to do daily tasks (*P* =.03). A greater proportion of participants reported “minimal” or no change in improvement in physical activity compared to overall status (71% vs. 52%, *P* =.01). Also, a smaller proportion of participants reported “much improvement” in the ability to do daily tasks (25% vs. 32%) and a greater proportion of participants reported “no change” (30% vs. 16%) compared to overall status. The association between overall PGIC status and change in PIS was slightly stronger for the SMART study sample compared to the IMPPPORT sample (*r* = 0.6 vs. *r* = 0.4, *P* =.09), but the difference was not statistically significant.

Table 3 shows pre-treatment PIS, post-treatment PIS, mean and percentage of PIS change during treatment, and mean PIS change by self-reported change in overall status for the combined sample as well as stratified by study and by baseline PIS (mild, moderate or severe). For the combined sample, the average change in PIS was a decrease of 2.7 points, and the average percentage change was a decrease of 8.5%. These changes represented a statistically significant decrease (at *P* <.05). The mean change and percentage change in PIS did not differ between studies. The mean change in PIS was smaller for the group with a mild baseline PIS compared to the groups with moderate or severe baseline PIS; similarly, the mean percentage change was smaller for the mild compared to the moderate PIS group.

**Table 3:** Responsiveness and minimal important change in Pain Impact score, by study and baseline impact score

	Study		Category of baseline PIS				
	Imppport	Smart	Mild (8–27)	Moderate (28–34)	Severe (35–50)	Overall	
Participants, *n*	61	192	107	89	57	253	
Scores, mean (SD)							
Pre-treatment PIS	26.4 (6.0)	29.9 (7.0)	22.6 (3.3)	30.7 (1.9)	38.7 (3.4)	29.1 (6.9)	
Post-treatment PIS	23.9 (6.1)	27.1 (8.8)	21.4 (6.2)	27.1 (6.1)	34.6 (8.1)	26.4 (8.3)	
Mean change in PIS	−2.4 (6.2)	−2.8 (6.4)	−1.2 (5.8)	−3.6 (6.0)	−4.0 (7.4)	−2.7 (6.4)	
Percentage change in PIS	−7.1 (23.7)	−9.0 (22.4)	−4.7 (26.0)	−11.8 (19.4)	−10.5 (19.8)	−8.5 (22.7)	
Mean change in PIS by self-reported overall status							
Very much improved	-10 (5.9)	-10 (7.7)	-7.1 (7.1)	-10 (4.6)	-19 (1.4)	-9.9 (6.7)	
Much improved	-3.3 (5.4)	-6.7 (5.5)	-3.6 (4.9)	-7.7 (4.6)	-10.9 (6.3)	-5.8 (5.6)	
Minimally improved	-0.9 (5.2)	-2.5 (5.0)	0.8 (4.1)	-3.4 (4.2)	-4.8 (5.6)	-2.2 (5.1)	
No change	-0.3 (6.1)	1.5 (5.1)	2.4 (5.8)	1.1 (3.7)	-0.3 (6.0)	1.2 (5.3)	
Minimally worse	2.6 (6.2)	1.4 (4.2)	2.8 (7.2)	4.4 (3.6)	-0.4 (3.2)	1.8 (4.7)	
Much worse		5.6 (5.2)	4	8 (7.1)	4.8 (5.7)	5.6 (5.3)	
MIC ROC approach							
Participants, *n*	61	189	106	88	56	250	
Cutpoint	-2.5	-4.5	-0.5	-4.5	-8.5	-2.5	
95% CI	-5.5, 0.5	-7.1, -1.9	-2.1, 1.1	-6.4, -2.6	-14.5, -2.4	-5.0, 0.0	
AUC	0.67	0.74	0.75	0.78	0.77	0.71	
Sensitivity	68%	69%	81%	77%	75%	76%	
Specificity	67%	79%	70%	79%	80%	66%	
MIC predictive modeling approach							
Cutpoint	-3.5	-3.8	-1.3	-4.7	-7.2	-3.4	
95% CI	-15.2, -0.1	-9.4, -1.2	-5.3, 0.7	-8.5, -2.5	-12.3, -4.4	-10.1, -0.7	
% much or very much improved	49%	36%	50%	35%	21%	38%	

Legend: AUC = area under the curve; CI, confidence interval; MIC = minimal important change; PIS, pain impact score; ROC = receiver operating characteristic. Cutpoint from Euclidean distance method (nearest to 0,1) displayed. Anchor for ROC analysis is PGIC item for patient-reported improvement in overall status

**Fig. 2:**
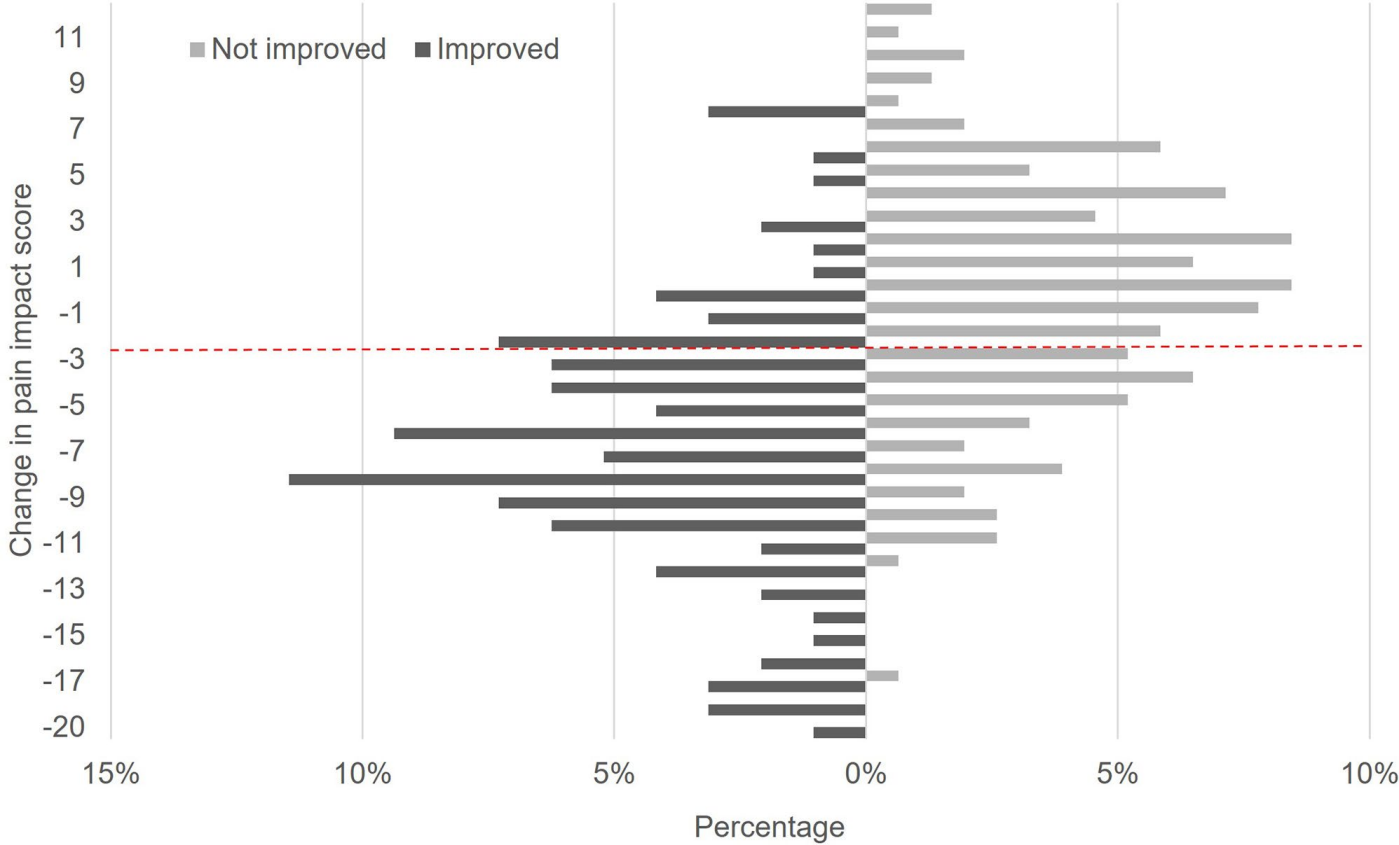
Distribution of change in pain impact scores above and below the minimal important change of 3 for patients that did and did not report improvement in overall status

Results from the ROC and predictive modeling analyses are shown in Table 3. The overall recommended MIC for the combined sample was a decrease of 3 in PIS (2.5 for ROC analysis and 3.4 for the predictive modeling approach). Figure 2 displays the distribution of PIS change scores above and below the MIC of 3 for participants who did and did not report important improvement. ROC curve is provided in Supplemental Fig. 1. The sensitivity and specificity for the ROC analysis were 76% and 66% respectively. With the exception of the MIC specific to the IMPPPORT study subgroup, all AUCs exceeded 0.70. The MIC estimates were smaller for those with mild baseline PIS and larger for those with moderate or severe baseline PIS. The MIC estimates were smaller in the IMPPPORT study sample than in the SMART study sample. For the SMART study, using pain intensity, ability to engage in physical activities, or ability to do day-to-day tasks as anchors—as compared to improvement in overall status—did not result in notably different MIC estimates.

Statistical measures used to assess significance of individual change were as follows: Standard Deviation Index = 14, Standard Error of Measurement = 7, Standard Error of Estimation = 6, Standard Error of Prediction = 9, and Coefficient of Repeatability (individual) = 9.1. The Coefficient of Repeatability for group level change was 0.6 [9]. 

## Discussion

The key finding from this analysis was that when using CAT versions of PROMIS physical function and pain interference, the PIS MIC corresponding to “much” or “very much” improvement was a decrease of about 3 points, depending on statistical approach (2.5 for ROC vs. 3.4 for predictive modeling). Given our observation of 38% of participants who reported more than minimal change and given the bias introduced in the ROC approach when the percentage is not equal to 50%, 3.4 is likely the more precise estimate. Statistics for significance of individual change were notable higher, ranging from 6 to 14 indicating that a larger change in PIS score is needed to confirm that an individual patient’s change is not due to measurement error. The MIC estimate varied substantially by baseline impact score, demonstrating that patients with greater initial pain-related limitations may need to experience greater improvements in pain intensity, pain interference and/or physical function before meeting the threshold for important change in overall status. This dependence of MIC on baseline values may support the use of different methods for categorizing important improvement, such as meeting a specified threshold (e.g., decreasing from moderate or severe pain impact to mild impact). The MIC also differed between the two study samples, likely due at least in part to a higher mean impact score in the SMART study sample.

A continuing challenge in estimating the PIS MIC is the lack of consensus on preferred methodology, such as (a) which anchor is assessed (e.g., pain, overall status); (b) number and categories of improvement options offered; and (c) threshold at which change is perceived as “important” (e.g., “minimal” vs. “much” improvement) [9]. Table 4 summarizes these differences in previously published MIC estimates. Overall, our MIC estimates are consistent with previous estimates, which range from 3 to 7.5: Our estimate mirrors that of Deyo et al., who estimated the MIC at 3, using a 5-option scale in an older population of rural primary care patients [4], but is somewhat lower than that of Dutmer et al., who estimated the MIC at 7.5 in a population with chronic low back pain cared for in a Dutch spine center [10]. In similar findings, Hays et al. estimated a MIC of 7 in a military population seeking chiropractic care for low back pain of any duration, using a 7-item categorical scale; unlike other studies, the scale used by Hays et al. included the option of “moderately improved.” [2].

**Table 4:**
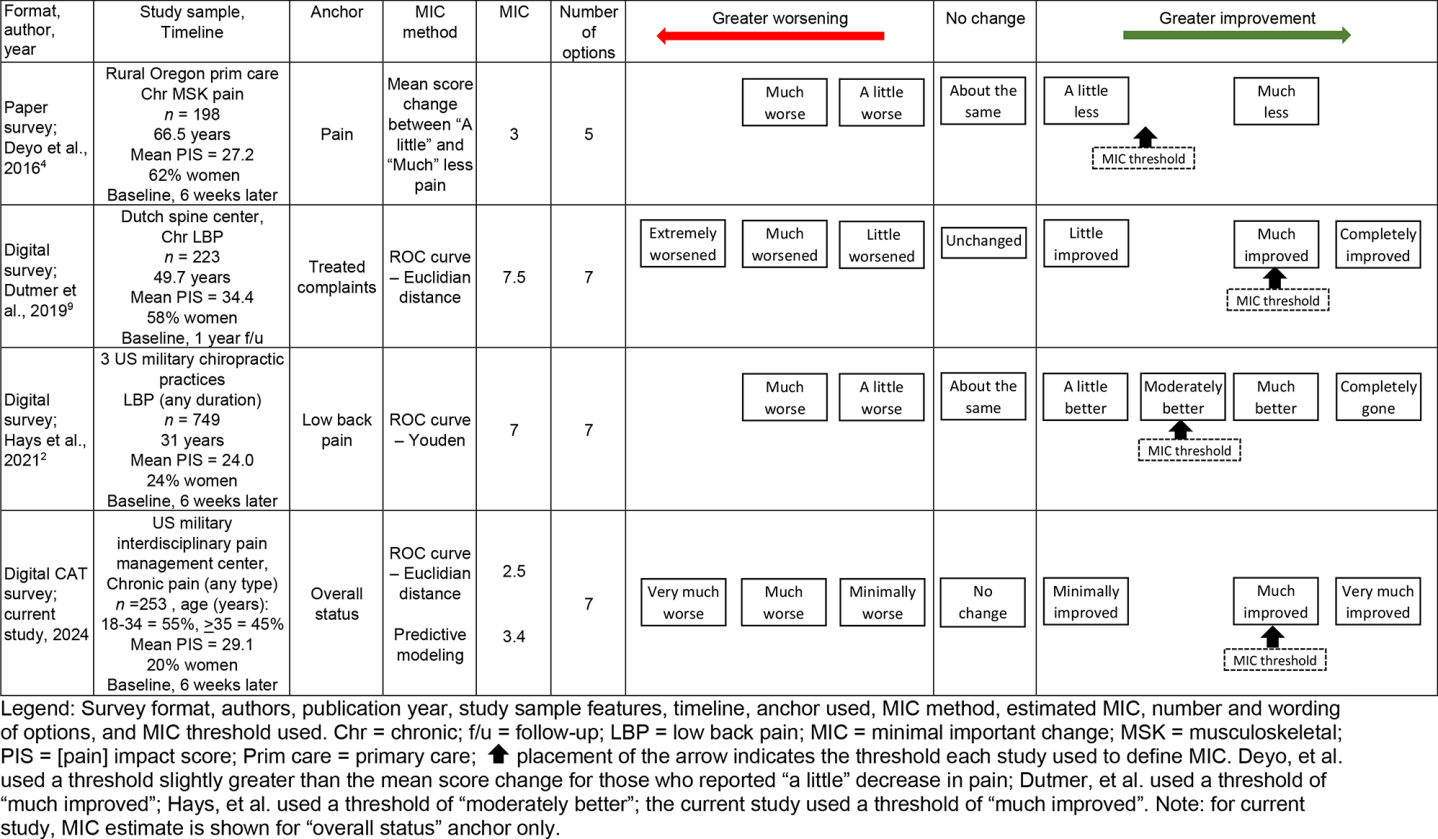
Prior research that estimated minimal important change in pain impact score

Our study has important implications particularly for researchers who want to examine PIS MIC using CAT versions of PROMIS measures. Researchers proposing to use PIS as an outcome measure can consider between-group decrease of at least 3–7 points to indicate important group-level difference, but should be aware that individual change scores of 14 or fewer points may be due to measurement error and should therefore be interpreted with caution. Additional research is needed to determine if using a specific PIS cutpoint, such as scores in the “mild” range (i.e., equal to or less than 27 points [1]), rather than an absolute change in PIS, is a better approach to defining response. In clinical practice, our findings can guide clinicians in determining response to treatment and making decisions on whether current treatment should be continued or modified.

A limitation of this study is that the 95% confidence limits for the overall MIC estimates yielded by the ROC and predictive modeling approaches included ranges from no change to 10.1 points improvement, thus future use of MIC estimates should also consider population characteristics and baseline PIS. In addition, our sample included only active-duty service members, and thus may not be representative of nonmilitary populations. Also, we used the DVPRS rather than the PROMIS pain intensity scale to compute PIS, which may have had modest effects on the impact score.

Other limitations include the lack of consensus about whether anchor-based MICs should be used to identify individual responders to treatment. Terwee and colleagues [8] suggest that MIC estimates can be used to identify responders to treatment. Conversely, Hays and Peipert [9] argue that MIC thresholds should not be used to identify responders to treatment because MIC estimates are averages derived from distributions of individual MICs and therefore may not reflect the perceived change by a given individual. Hays and Peipert recommend presenting both individual statistical significance and whether the individual feels they have improved to identify responders to treatment. In addition, Hays and Peipert note that including all those who change rather than focusing on those with minimal but important change can result in the MIC threshold estimates that are too large.

## Conclusions

We used the CAT versions of PROMIS physical function and pain interference measures to obtain an estimate of the PIS MIC that was similar to those reported in previous studies that used PROMIS short-form measures. This similarity opens new options for researchers and clinicians when assessing pain treatment response to use CAT questionnaires, which have greater accuracy than short-form questionnaires.

## Electronic supplementary material

Below is the link to the electronic supplementary material.

Supplementary Material 1: Supplemental Fig. 1. Receiver operating characteristic curve for impact score change cutpoint associated with “much improved” or “very much improved” overall status

## Data Availability

The current approved research protocol does not permit sharing of data that were used for this analysis. However, reasonable requests for de-identified data to the corresponding author may be considered for release following an IRB-approved protocol modification.

## Declarations

### Disclaimer

The views expressed are those of the authors and do not reflect the policy or position of the Department of the Army, Department of Defense, the National Institutes of Health or the US Government. The content is solely the responsibility of the authors. The investigators adhered to the policies for protection of human subjects as prescribed in 45 CFR 46.

## Abbreviations

AUC: Area under the curve
CAT: Computer adaptive testing
CI: Confidence interval
CUTPT: Module to determine optimal cutpoint in Stata statistical software package
DVPRS: Defense and Veterans Pain Rating Scale
GED: General education diploma
ICD-10: International classification of diseases, 10th edition
IMPPPORT: Integrative Modalities Plus Psychological, Physical and Occupational Restorative Therapies
IRB: Institutional review board
LBP: Low back pain
MIC: Minimal important change
NIH RTF: National Institutes of Health Task Force on Research Standards for Chronic Low Back Pain
PGIC: Patient global impression of change
PIS: Pain impact score
PROMIS: Patient-Reported Outcomes Measurement Information System
RCI: Reliable change index
RCT: Randomized clinical trial
ROC: Receiver operating characteristic
SMART: Sequential multiple assignment randomized trial
US: United States
