# Supplementary figures and images for: Estimating minimal important change of the National Institutes of health research task force impact score using computer adaptive measures: a secondary analysis of two randomized clinical trials in a military population with chronic pain
Source: BMC Musculoskelet Disord. 2025 Feb 11;26:137. doi: 10.1186/s12891-025-08378-5 (PMC11817800; doi:10.1186/s12891-025-08378-5)

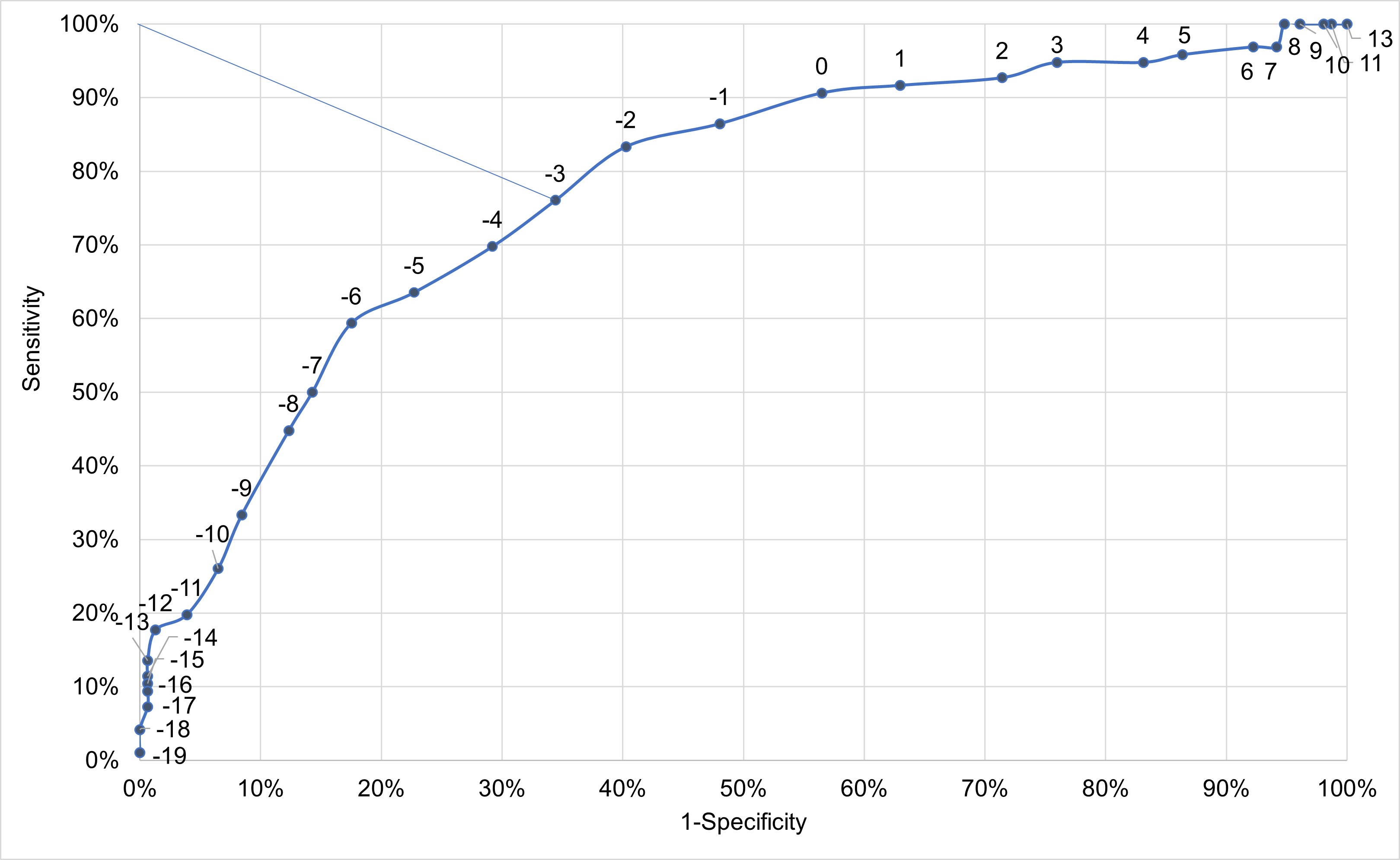

Supplement: Supplementary file 1 — Supplementary Material 1: Supplemental Fig. 1. Receiver operating characteristic curve for impact score change cutpoint associated with “much improved” or “very much improved” overall status [file 12891_2025_8378_MOESM1_ESM.jpg]
